# Monoterpene-Rich Nanoemulsion from *Thymus vulgaris* as a Promising Acaricidal Strategy Against *Tetranychus mexicanus*: Effects on Survival and Fecundity

**DOI:** 10.3390/molecules31122167

**Published:** 2026-06-20

**Authors:** Geraldo J. N. Vasconcelos, Raul V. C. Apolinário, Tatiane M. S. Cardoso, Jefferson D. Cruz, Walter S. M. F., Maria A. Mpalantinos, Jefferson R. A. Silva, Ana Claudia F. Amaral

**Affiliations:** 1 Instituto de Ciências Exatas e Tecnologia (ICET), Universidade Federal do Amazonas, Itacoatiara 69103-128, AM, Brazil; gjnvasconcelos@ufam.edu.br (G.J.N.V.); tatiane.motta@hotmail.com (T.M.S.C.); 2 Laboratório de Plantas Medicinais e Derivados, Farmanguinhos, ITC, Fundação Oswaldo Cruz, Rio de Janeiro 21041-250, RJ, Brazil; apolinario_raul@yahoo.com (R.V.C.A.); jefferson_dacruz@hotmail.com (J.D.C.); maria.mpalantinos@fiocruz.br (M.A.M.); 3 Laboratório de Cromatografia, Departamento de Química, Instituto de Ciências Exatas, Universidade Federal do Amazonas, Manaus 69077-000, AM, Brazil; wssottoo@gmail.com

**Keywords:** mite, Lamiaceae, terpenes, nanoformulation, morphology, molecular docking

## Abstract

Mounting acaricide resistance in *Tetranychus mexicanus* (McGregor) (Acari: Tetranychidae), among the most damaging phytophagous mites in tropical and subtropical crops, has intensified the search for botanical alternatives. An oil-in-water nanoemulsion of *Thymus vulgaris* essential oil (TVEO-NE) was developed and evaluated for lethal and sublethal effects on adult females of *T. mexicanus*. TVEO, composed mainly of thymol (45%) and p-cymene (37%), was formulated by low-energy emulsification yielding stable dispersions (~200 nm; PDI < 0.25; zeta potential of −22.2 mV). At 30.0 mg a.i./mL, TVEO-NE caused 68.3% corrected mortality at 72 h and suppressed fecundity by ~44–52%; vehicle controls exerted only moderate effects, identifying the essential oil as the primary bioactive driver. Morphological examination revealed collapse of female idiosomata and disruption of excretory pellet architecture, corroborating the bioassay data. Molecular docking against a cathepsin L homology model revealed that thymol and p-cymene interact exclusively via hydrophobic contacts and display substantially lower ChemPLP fitness scores than the reference cysteine protease inhibitor E64, indicating weak predicted binding affinity and arguing against enzyme inhibition as the primary mechanism. Taken together, bioassay, morphological, and docking are consistent with supporting membrane partitioning as a plausible primary mode of action, positioning TVEO-based nanoemulsions as promising botanical tools for *T. mexicanus* management.

## 1. Introduction

Mites of the family Tetranychidae represent a persistent agricultural threat, recognized by their web-spinning behavior and their capacity to colonize ornamental plants and fruit trees across diverse agroecosystems [1]. Direct feeding on plant cells provokes chlorosis, premature leaf abscission, and, under severe infestation pressure, plant death [2]. The silken web produced by these mites shelters immature developmental stages from both natural enemies and unfavorable climatic conditions, facilitating colony establishment and long-term persistence on host plants [3].

Within the Tetranychidae, *Tetranychus mexicanus* (McGregor, 1950), commonly referred to in the literature variously as the Mexican mite or red mite, has emerged as an increasingly important pest throughout the Americas, with significant economic repercussions across the Amazon Basin [4,5]. The species exploits an unusually wide host range, causing economically significant losses on passion fruit, cocoa, cotton, banana, citrus, papaya, pear, strawberry, soursop, and guaraná [5,6]. A closely related species, *T. urticae*, causes analogous damage on cucurbits, solanaceous crops, and various field crops; at high population densities, both species can rapidly desiccate host tissues, reducing photosynthetic capacity and ultimately compromising crop yield [7,8]. Despite its expanding geographic distribution and clear relevance to tropical fruit production, *T. mexicanus* has attracted substantially less scientific attention than *T. urticae*, which remains the most extensively studied tetranychid globally.

The intensification of agricultural production, compounded by climate-related environmental changes, has contributed to increased mite prevalence and escalating control costs [9,10]. Reliance on synthetic acaricides, though initially effective, has generated documented resistance, reduced field efficacy over time, ecosystem contamination, and adverse impacts on non-target fauna [11]. Resistance development in *Tetranychus* spp. is mechanistically well-understood, driven by short generation times, high fecundity, and arrhenotokous reproduction traits that collectively accelerate selection under repeated chemical exposure [9]. These constraints have directed research efforts toward alternative strategies, including biological control with predatory Phytoseiidae, the use of entomopathogenic fungi such as *Beauveria bassiana* and *Metarhizium anisopliae*, and plant-derived compounds, including essential oils from various species, all of which have shown acaricidal potential [12,13,14].

Essential oils are complex volatile mixtures of plant secondary metabolites, predominantly monoterpenes, sesquiterpenes, and phenylpropanoids obtained from leaves, stems, flowers, or roots [15,16]. In addition to their established roles in human health, food preservation, and perfumery, essential oils exert a range of effects on arthropods, including repellency, disruption of larval development, oviposition deterrence, antifeedant activity, and direct mortality [17,18]. Species from the genera *Origanum*, *Lippia*, *Salvia*, *Thymus*, *Dysphania*, *Clausena*, *Mangifera*, and *Citrus* are frequently cited in the acaricidal literature [19,20,21,22,23]. The family Lamiaceae, encompassing approximately 230 genera and 7100 species, includes several of these bioactive taxa, notably *Salvia rosmarinus*, *Origanum vulgare*, and *Thymus vulgaris*, which are currently under active investigation for pest management applications [23,24].

*Thymus vulgaris* L. (thyme) warrants specific consideration in this context. The essential oil of *T. vulgaris* and its isolated constituents have demonstrated acaricidal activity against tetranychid mites, including *Tetranychus urticae* (LC_50_ = 1.84 µL/100 mL air [25]). The principal phenolic monoterpenoid, thymol, accounts for much of this activity, acting both individually and in synergy with minor constituents such as p-cymene and γ-terpinene [26]. Beyond acaricidal efficacy, thyme essential oil exhibits larvicidal, antimicrobial, and anti-inflammatory properties, which support its broader applicability within integrated pest management frameworks [27,28]. Nevertheless, the unformulated essential oil presents inherent physicochemical limitations in practical field application: rapid volatilization leads to premature dissipation, low water solubility impairs homogeneous spray distribution, and susceptibility to oxidation reduces residual activity [29]. To address these constraints, oil-in-water nanoemulsions have been developed as delivery systems intended to improve physicochemical stability, enhance foliar adhesion, and extend biological efficacy at reduced concentrations of active ingredient [20,21,30].

Given that *T. mexicanus* remains inadequately characterized as a pest target despite its growing impact on tropical production systems, and that dedicated acaricidal tools compatible with integrated management are scarce, we developed and physicochemically characterized an oil-in-water nanoemulsion of *T. vulgaris* essential oil stabilized with Tween 20. Lethal and sublethal effects on adult females were evaluated concurrently, allowing us to distinguish the biological contributions of the essential oil from those of the surfactant vehicle. Additionally, homology modeling and molecular docking of the principal TVEO constituents were performed against cathepsin L, a digestive cysteine protease in tetranychid mites, to investigate whether protease inhibition mechanistically contributes to the observed toxicity. The experimental and computational data presented here are intended to provide a rational basis for the application of botanical nanoemulsions in the management of *T. mexicanus*.

## 2. Results and Discussion

### 2.1. Chemical Profile and Physicochemical Characterization of TVEO-NE

Hydrodistillation of *T. vulgaris* leaves yielded 0.3% (*w*/*w*) essential oil, obtained as a slightly yellow liquid with a characteristic spicy–herbal odor. Analysis of the GC–MS chromatogram obtained from the essential oil of *T. vulgaris* showed thymol as the major constituent (45.86%), followed by p-cymene (37.0%) (Table 1). The total identified fraction corresponded to 98.56% of the essential oil composition. This composition is consistent with the thymol/p-cymene chemotype previously documented for *T. vulgaris* [31,32,33]. The predominance of thymol and p-cymene, together accounting for 82.9% of the total composition, provides the phytochemical framework for interpreting the biological activity data reported in the following sections, including the previously reported sublethal effects of *T. vulgaris* essential oil on *T. urticae* [34].

#### Formulation and Physicochemical Properties of TVEO-Nanoemulsion (NE)

TVEO-NE exhibited macroscopic stability and was therefore advanced to physicochemical characterization and bioassays (Table 2). The mean droplet size of 200.70 ± 8.9 nm with HLB 16.7 yielded a physically stable nanoscale dispersion, consistent with the typical range reported for essential oil systems based on Lamiaceae species prepared with polysorbate surfactants [35,36]. Al-Asmary and coworkers [36] reported analogous droplet sizes for oregano (~185 nm) and thyme (~130 nm) essential oil nanoemulsions in antimicrobial studies, confirming that the values obtained here fall within the typical range for stable Lamiaceae essential oil systems.

The low polydispersity index (PDI 0.237) indicated a narrow droplet-size distribution and adequate formulation homogeneity [28]. PDI values below ~0.25 are frequently interpreted as indicative of relatively homogeneous nanoemulsions and essential oil nanoemulsions stabilized with polysorbates often fall within this interval when formulation and processing conditions are well controlled [35,36,37]. The zeta potential of TVEO-NE was −22.2 ± 0.9 mV, indicating a kinetically stable system in which inter-droplet electrostatic repulsion and steric stabilization provided by the non-ionic surfactant contributed to colloidal integrity [36]. The Blank-NE displayed similar physicochemical parameters, demonstrating that incorporation of TVEO did not compromise colloidal stability.

The combination of nanometric droplet size, low PDI, and moderately negative ζ-potential is consistent with formulations expected to spread on the leaf surface and to improve contact between hydrophobic actives and the mite cuticle, while retaining physical stability during the bioassay window [38].

Mossa et al. [21] developed a rosemary essential oil nanoemulsion stabilized with polysorbate surfactants, with a mean particle size of 139.9 nm. Similarly, Badawy et al. [20] prepared nanoemulsions from *Callistemon viminalis* and *Origanum vulgare* essential oils using high-energy emulsification, obtaining nanosystems with particle sizes between 7 and 10 nm and PDI values ranging from 0.249 to 0.620. The physicochemical parameters observed for TVEO-NE, including a droplet size of 200.70 ± 8.9 nm and a PDI of 0.237, are therefore consistent with values reported for stable botanical essential oil nanoemulsions developed for acaricidal applications.

### 2.2. Mortality of Tetranychus Mexicanus Exposed to TVEO-NE

Corrected mortality was evaluated at 24, 48, and 72 h to characterize the time-kill profile of TVEO-NE. Because raw natural mortality in the untreated control was above 5% but below 20%, mortality data were corrected according to Abbott/Schneider-Orelli. Factorial ANOVA indicated strong main effects of both exposure time and formulation, as well as a significant formulation × time interaction (Table S1). This interaction supports a formulation-dependent time course, i.e., the relative advantage of TVEO-NE over Blank-NE changes across the assay window.

**Figure 1:**
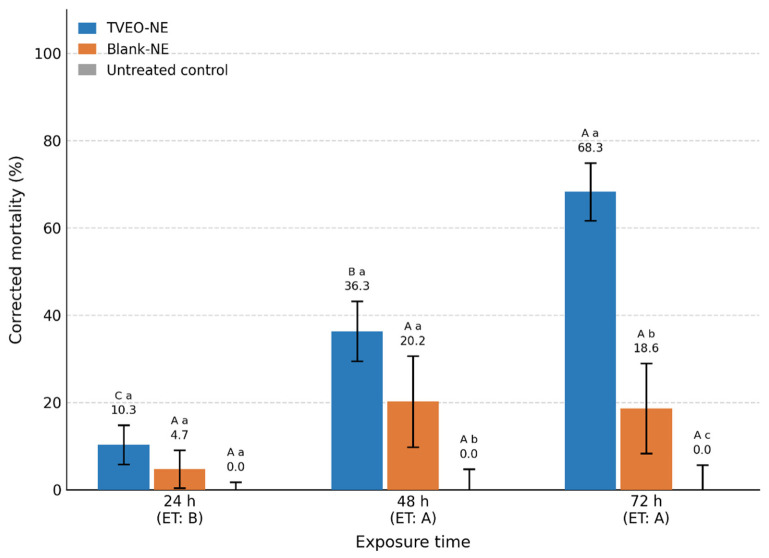
Corrected mortality (%) of adult females of *Tetranychus mexicanus* at different exposure times to TVEO-NE. Blue bars represent the active nanoemulsion (TVEO-NE); orange bars represent blank-NE (excipient control); gray indicates the untreated control (corrected mortality = 0%, reference baseline). Values are shown as mean ± SEM. Means followed by the same letter (uppercase within rows and lowercase within columns) do not differ by the Scott–Knott test (*p* ≤ 0.05). ET indicates the exposure-time factor.

TVEO-NE caused a progressive increase in mortality from 10.3 ± 4.46% at 24 h to 36.3 ± 6.86% at 48 h and 68.3 ± 6.61% at 72 h (Figure 1). Over the same period, Blank-NE induced low to moderate mortality, with values ranging from 4.7 ± 2.55% to 20.2 ± 5.01%, indicating that the vehicle itself contributes to contact stress but does not account for the pronounced 72 h mortality reached by TVEO-NE. The untreated control is shown as 0% corrected mortality throughout the assay because it served as the correction baseline, not because raw natural mortality was absent. The magnitude of mortality and its progressive kinetics indicate that the essential oil was the main acaricidal driver, with thymol and p-cymene, together representing 81.9% of the EO, likely serving as the principal active constituents, in agreement with the well-established membrane-partitioning toxicity of monoterpenes [26,39].

The acaricidal efficacy observed here is consistent with gains reported for other botanical nanoemulsions against tetranychid mites. Mossa et al. [21] documented toxicity increases of 54% and 53% over crude rosemary essential oil for immature and adult stages of *T. urticae*, respectively, with no mammalian toxicity in rats. Badawy et al. [20] achieved high acaricidal efficacy against *T. urticae* without phytotoxicity using *Origanum vulgare* nanoemulsions, reinforcing the advantage of nanoemulsion delivery over free essential oils.

### 2.3. Inhibition of Fecundity and Sublethal Reproductive Effects

Sublethal effects were assessed as fecundity inhibition, defined as the reduction in oviposition relative to the untreated control, at the same exposure times evaluated for mortality. ANOVA revealed a significant effect of formulation on fecundity inhibition (*p* < 0.0001), whereas neither exposure time (*p* = 0.5685) nor the formulation × time interaction reached significance (*p* = 0.4982) (Table S2).

**Figure 2:**
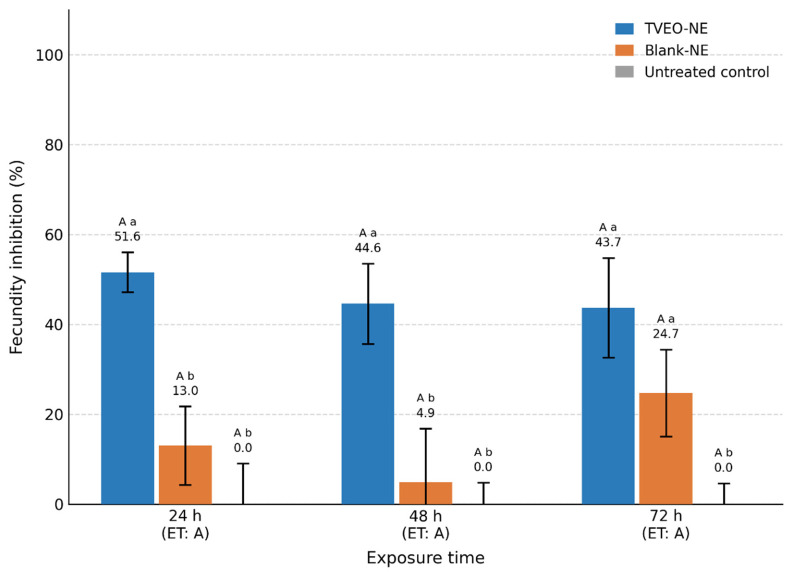
Fecundity inhibition (%) of *Tetranychus mexicanus* at different exposure times to TVEO-NE. Blue bars represent the active nanoemulsion (TVEO-NE); orange bars represent blank-NE (excipient control); gray indicates the untreated control (fecundity inhibition = 0%, reference baseline). Values are shown as mean ± SEM. Means followed by the same letter (uppercase within rows and lowercase within columns) do not differ by the Scott–Knott test (*p* ≤ 0.05). ET indicates the exposure-time factor.

These results indicate that the reduction in oviposition was driven primarily by the treatment itself and remained relatively stable throughout the 24–72 h observation period. Consistent with this pattern, TVEO-NE suppressed fecundity by approximately 44–52% at all evaluated time points (*p* ≥ 0.4578), reaching 51.6% at 24 h, 44.6% at 48 h, and 43.7% at 72 h (Figure 2). By contrast, Blank-NE produced only limited and inconsistent inhibition, with values of 13.0%, 4.9%, and 24.7% at 24, 48, and 72 h (*p* ≥ 0.0563), respectively, reinforcing that the vehicle alone does not completely explain the reproductive suppression induced by TVEO-NE. Taken together, the modest effects of the blank formulation and the consistent fecundity suppression produced by TVEO-NE across all exposure times indicate that the essential oil is the main driver of the sublethal reproductive effect (Figure 2).

These results show that even when mortality is still moderate (≤36% at 48 h), TVEO-NE already imposes substantial reproductive suppression, a key consideration for population growth control in *Tetranychus* spp., where rapid oviposition drives outbreaks. The modest but non-negligible fecundity inhibition recorded for Blank-NE (13.0–24.7%) is consistent with evidence that surfactant co-formulants can independently suppress reproduction-related endpoints in tetranychid bioassays without proportional lethality, as documented for organosilicone adjuvants in *T. urticae* [40]. This vehicle contribution, while limited relative to the TVEO-NE effect, reinforces that surfactant load should be treated as an explicit design variable in future assays, and that fecundity inhibition data should always be interpreted in relation to matched vehicle control. The mechanisms underlying the residual activity of Blank-NE most plausibly involve physicochemical rather than target-specific biochemical effects. In leaf-disc bioassays with *Tetranychus* spp., surfactants such as Silwet L-77 have been used to reduce surface tension and promote the spreading of aqueous test solutions over the leaf surface; however, this co-formulant has also been shown to affect mite biology, reducing fecundity and feeding activity in *T. urticae* even when survival was not significantly reduced [40]. The residual effects observed for Blank-NE may therefore reflect surfactant-associated physical stress and/or behavioral interference, including altered spreading of residues on the treated leaf surface, increased contact with the mite body surface, and possible ingestion of surfactant residues during feeding. Consistent with this interpretation, Abouelmaaty and coworkers suggested that Silwet L-77 delivered to the midgut of *T. urticae* may inhibit feeding and thereby impair normal fecundity [40]. Because the present Blank-NE contains polysorbate 20 rather than an organosilicone surfactant, whether analogous effects occur through comparable mechanisms remains to be directly established. Nevertheless, the matched Blank-NE control shows that the vehicle contribution was moderate and did not account for the stronger, time-progressive lethality and consistent fecundity suppression induced by TVEO-NE.

More broadly, the recognition that sublethal exposure, whether to active compounds or to co-formulants, can profoundly alter arthropod reproductive behavior independently of adult lethality [41] highlights the importance of quantifying oviposition inhibition as a complementary endpoint in acaricide evaluations.

### 2.4. Morphological Observations on Treated Colonies

**Figure 3:**
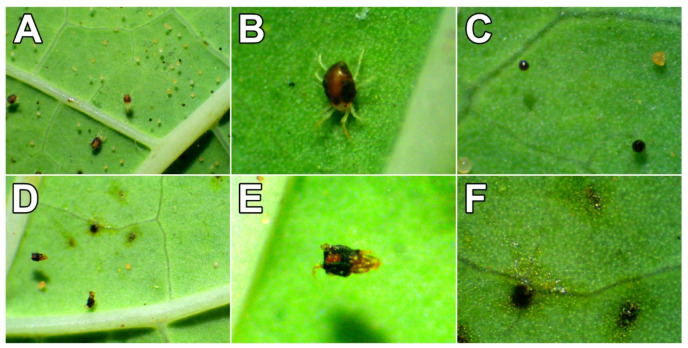
Qualitative effects of *Thymus vulgaris* essential oil nanoemulsion (TVEO-NE) on *Tetranychus mexicanus* colonies on papaya leaf discs: (**A**–**C**) untreated control; (**A**) colony with healthy adult females; (**B**) detail of a feeding female; (**C**) normal fecal pellets retained in the web above the leaf surface. (**D**–**F**) colonies treated with TVEO-NE for 24 h; (**D**) colony with dead females showing a dehydrated appearance; (**E**) detail of a dead female with collapsed idiosoma; (**F**) excreta without discrete pellet formation, appearing as aqueous smears on the leaf surface.

Spider mites of the genus *Tetranychus* produce abundant silk on the leaf surface, which protects eggs, immature stages, and spherical fecal pellets that are either black and solid or yellow and viscous. Classic studies identified guanine as a major nitrogenous waste in spider mites and documented multiple excretory products [42,43,44]. In untreated controls, *T*. *mexicanus* colonies on papaya discs displayed actively feeding females, abundant eggs, and normal pellet formation within the webbing. In contrast, colonies exposed to TVEO-NE for 24 h showed dead females with collapsed idiosomata, fewer eggs, and excreta that no longer formed discrete pellets but instead appeared as aqueous smears, in agreement with the quantitative mortality and fecundity data (Figure 3).

#### 2.4.1. Structural Model Quality and Validation

Comparative quality assessment of three homology modeling approaches revealed notable differences in model reliability (Table 3). Among the tested platforms, the SWISS-MODEL structure exhibited the most consistent overall stereochemistry, as indicated by the lowest MolProbity score (1.49), clashscore (0.37), and a ProSA Z-score of −7.57 [45,46], while maintaining a high fraction of residues in favored Ramachandran regions (92.84%) and a low outlier rate (2.29%). Side-chain quality was also acceptable (3.33% rotamer outliers; 4 Cβ deviations). In comparison, the Phyre2.2 model showed equivalent global quality scores (QMEAN6 0.734; Z-QMEAN −1.06; ProSA Z-score −7.79) [46,47,48] and slightly improved backbone statistics (93.97% Ramachandran favored; 1.27% outliers) with no rotamer outliers; however, its elevated clashscore (50.08) and higher MolProbity score (2.58) indicate steric overlaps [45], suggesting that additional refinement would be required prior to downstream applications. The I-TASSER model presented the worst overall performance across all metrics, with a MolProbity score of 5.11, a clashscore of 361.45, and sharply reduced global quality (QMEAN6 0.439; Z-QMEAN −8.32; ProSA Z-score −8.09) [46,47,48], accompanied by poor stereochemical features (76.22% Ramachandran favored; 9.17% outliers; 54.33% rotamer outliers; 313 Cβ deviations).

These results support the SWISS-MODEL-derived structure as the preferred starting model for subsequent computational analyses. The strong performance of SWISS-MODEL is consistent with the established reliability of template-based homology modeling when suitable templates are available [49]. The QMEAN6 value of 0.734, exceeding the commonly accepted threshold of 0.70 for high-quality models, and the favorable stereochemical parameters provide a robust computational foundation for molecular docking studies. The selection of SWISS-MODEL is further justified by the AlphaFold v2 template used (T1KVN5.1.A), which achieved 100% sequence identity and coverage across all 351 residues, effectively representing a direct structural transfer with minimal modeling uncertainty.

#### 2.4.2. Molecular Docking of Tveo Principal Constituents Against Cathepsin L

The acaricidal and antifecundity effects observed in Section 2.2, Section 2.3 and Section 2.4 may reflect combined cuticular and oral exposure. During the leaf disc bioassay, *T. mexicanus* females feed by piercing the leaf epidermis with cheliceral stylets and ingesting plant cell contents, a process during which nanoemulsion droplets deposited on the adaxial surface are likely co-ingested. Bensoussan et al. [50] demonstrated that fluorescent microspheres ranging from 50 to 500 nm accumulated in the midgut of *T. urticae* after 24 h of feeding. Since the nanoemulsions characterized in the presented study showed mean droplet sizes of approximately 200 nm (Table 2), they fall within this experimentally observed size range, supporting plausibility of oral uptake during feeding. Santamaría et al. [51] identified cathepsin L-like and aspartyl proteases as the predominant digestive proteases active in the midgut lumen at the acidic pH (3.5–5.5) typical of tetranychid digestive compartments. Direct experimental support for this pathway comes from El-Sayed et al. [52], who demonstrated that ingested anise essential oil (*Pimpinella anisum*) significantly inhibited both protease and acetylcholinesterase activity in *T. urticae* under bioassay conditions analogous to those used here.

**Figure 4:**
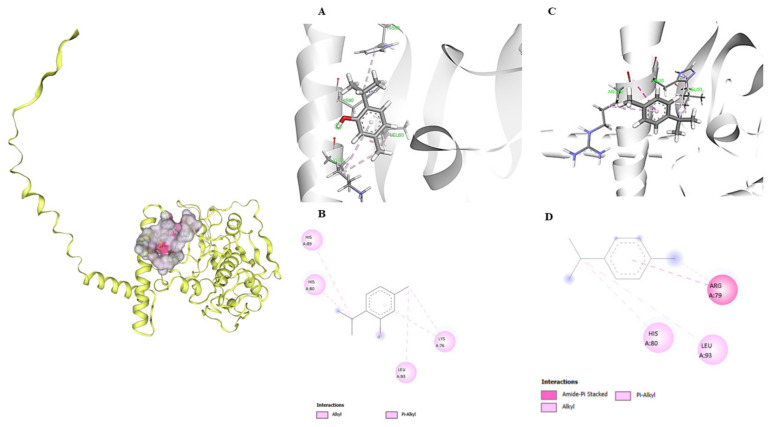
Intermolecular interactions of TVEO principal constituents with cathepsin L from *Tetranychus mexicanus*. Left panel: three-dimensional structure of cathepsin L with the binding site region highlighted (space filling representation). (**A**,**B**) Thymol: (**A**) three-dimensional view of the ligand cathepsin L complex; (**B**) two-dimensional diagram of intermolecular interactions. (**C**,**D**) p-cymene: (**C**) three-dimensional binding pose; (**D**) two-dimensional interaction diagram. Interaction legend: alkyl interactions (light pink), π-alkyl interactions (pink), and amide-π stacked interactions (magenta). Visualizations generated with BIOVIA Discovery Studio 2021.

Molecular docking was performed against the SWISS-MODEL cathepsin L homology model to investigate potential interactions between thymol and p-cymene, the principal TVEO constituents and this digestive target. E64 (trans-epoxysuccinyl-L-leucylamido-(4-guanidino) butane), a canonical irreversible cysteine protease inhibitor, served as positive control. Results are summarized in Table 4. Interaction maps for thymol and p-cymene are presented in Figure 4, whereas those for E64 and the Tween 20 surrogate are provided in Figure S1.

Thymol and p-cymene displayed highly convergent binding predictions (ΔFitness < 1.1), forming exclusively hydrophobic contacts, including alkyl and π-alkyl interactions, with HIS A:80, HIS A:89, LYS A:76, and LEU A:93 in the case of thymol (Figure 4A,B), and ARG A:79, HIS A:80, and LEU A:93 in the case of p-cymene (Figure 4B,C). This interaction pattern is consistent with the high lipophilicity of both compounds reflected by log P values of 3.3 and 4.0, respectively. The absence of conventional hydrogen bonds across all poses and the substantially lower PLP.Fitness scores relative to E64 (35–36 vs. 63.84) suggest hydrophobic occlusion of the substrate-binding region rather than substrate-mimetic inhibition, although partial inhibitory activity at concentrations achievable in the midgut lumen following nanoemulsion ingestion cannot be excluded [52].

The reference inhibitor E64 established multiple conventional hydrogen bonds (PLP.Chemscore.Hbond = 5.38) with GLU A:276, GLU A:288, GLU A:290, SER A:277, and PRO A:289 (Figure S1A), supporting the ability of the docking protocol to correctly position ligands within the catalytic cleft.

Tween 20 was included as an additional docking ligand because it is the main excipient of the formulation. Due to the structural complexity of commercial polysorbate 20 (average Mw ~1228 Da; heterogeneous polyoxyethylene chains), docking was conducted using the only chemically defined structural representative available in curated chemical databases (PubChem CID 443314; sorbitan monolaurate ethoxylate, Mw 482 Da), which represents a truncated analog of the intact surfactant approximately 2.5-fold smaller than the native polysorbate. The surrogate yielded a PLP.Fitness score of 69.20, nominally exceeding that of E64 and the monoterpenes. This comparison, however, is not structurally meaningful: the compact geometry and reduced conformational complexity of the surrogate relative to native polysorbate 20 are expected to artificially favor accommodation within a finite binding cavity, independently of true binding affinity. The result should therefore be interpreted as a structural approximation subject to substantial uncertainty, not as evidence of inhibitory potential of the polysorbate excipient. Accordingly, the biological contribution of the vehicle is interpreted primarily from the matched Blank-NE control in the bioassays, rather than from the docking score of the Tween 20 surrogate. Interaction maps for E64 and the Tween 20 surrogate are shown in Figure S1.

Taken together, the docking results do not support selective cathepsin L inhibition by thymol or p-cymene as the primary acaricidal mechanism. Instead, the convergent and exclusively hydrophobic binding pattern observed for both monoterpenes is more consistent with membrane partitioning as the main determinant of their bioactivity, likely acting systemically after absorption through the gut epithelium or across the cuticle. The convergence of the experimental and computational evidence is consistent with, but does not definitively establish, membrane partitioning as the primary mode of action of TVEO-NE. Definitive mechanistic attribution would require direct experimental support, including enzyme activity assays, gene expression data, membrane permeability assays, and ultrastructural analyses of the cuticle and gut epithelium, all of which are identified as priorities for follow-up investigation. The docking results are therefore best interpreted as a computational screening indicating that selective cathepsin L inhibition is an unlikely primary contributor to the observed toxicity, rather than as positive evidence for an alternative mechanism.

The present study integrates physicochemical formulation, bioassay, and computational analyses to establish TVEO-NE as a promising acaricidal formulation against *T. mexicanus*. The progressive increase in mortality over 72 h, ranging from 10.3–68.3%, together with the consistent inhibition of fecundity, approximately 44 to 52%, despite only moderate effects of Blank-NE, indicates that the essential oil, rather than the Tween 20 vehicle, is the main bioactive component responsible for the observed activity. This distinction is methodologically relevant, since surfactant-based blank formulations may themselves induce sublethal stress in Tetranychidae, as previously reported in surfactant-treated leaf-disc assays with *T. urticae* [38].

The molecular docking results provide a mechanistic counterpoint to the bioassay findings, as thymol and p-cymene showed low ChemPLP fitness scores (35–36, compared with 63.84 for E64) and interacted with cathepsin L exclusively through hydrophobic contacts, indicating that specific inhibition of this cysteine protease is unlikely to represent the predominant mode of action. This interpretation is consistent with the established toxicological behavior of monoterpenes, which is more commonly associated with membrane partitioning and cytotoxic disruption than with receptor-mediated inhibition [26,40,52]. The convergence of the experimental and computational evidence strengthens the conclusion that TVEO-NE exerts its acaricidal and antifecundity effects primarily through physicochemical disruption of the mite cuticle and gut epithelium.

#### 2.4.3. Molecular Dynamics Simulation of Cathepsin L Complexes

**Figure 5:**
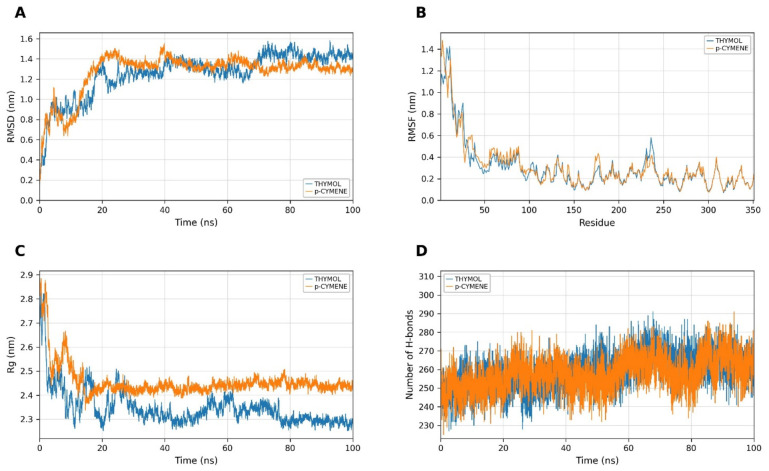
Molecular dynamics simulation of cathepsin L complexes with thymol and p-cymene over 100 ns. (**A**) Backbone root mean square deviation (RMSD), (**B**) root mean square fluctuation (RMSF) per residue, (**C**) radius of gyration (Rg), and (**D**) hydrogen-bond count. Both trajectories were collected after NPT equilibration; statistics reported in the text refer to the 80–100 ns production window.

Cathepsin L complexes with thymol and p-cymene underwent molecular dynamics simulations (MD) over 100 ns to evaluate the temporal stability of the docking predicted binding poses (Figure 5). Both systems underwent a rapid structural adjustment during the first 15–20 ns, after which the backbone RMSD stabilized and fluctuated within a narrow range for the remainder of the trajectory. Over the 80–100 ns interval, the p-cymene complex maintained a lower mean backbone RMSD than the thymol complex (1.32 ± 0.04 vs. 1.44 ± 0.04 nm), indicating that the p-cymene-bound enzyme deviated less from its initial conformation upon equilibration.

Residue level flexibility, as reflected by the RMSF profiles, showed two regions of increased mobility shared by both complexes: an N-terminal segment comprising approximately residues 1 to 20, with RMSF values reaching about 1.3 to 1.4 nm, and a central region around residues 150 to 160, where thymol reached values of about 0.55 to 0.60 nm. This latter segment likely corresponds to one of the R domain loops flanking the active site cleft of cathepsin L, a region known to exhibit context-dependent conformational variability [53]. In both complexes, the increase in RMSF around residue 150 was more evident in the thymol-bound system than in the p-cymene-bound system, indicating a slightly greater effect of thymol on this functionally relevant loop. Apart from these two more flexible regions, most residues showed RMSF values below 0.3 nm, supporting the maintenance of the overall tertiary structure throughout both simulations. The radius of gyration profiles differed clearly between the two ligand-bound systems (Figure 5C). After an initial expansion at the beginning of the simulations, with values close to 2.8 nm in both complexes, the thymol-bound protein showed a more pronounced reduction in Rg and gradually reached a mean value of 2.29 ± 0.01 nm during the last 20 ns. In contrast, the p-cymene complex displayed a less marked decrease and stabilized at a higher mean value of 2.44 ± 0.01 nm over the same period. These results indicate that thymol binding was associated with a greater overall compaction of cathepsin L, whereas the enzyme remained in a comparatively more expanded conformational state in the presence of p-cymene. Notably, this reduction in Rg in the thymol complex was not accompanied by any evident structural disruption throughout the trajectory.

Despite the distinct compaction profiles, the intramolecular hydrogen-bond networks of both complexes remained essentially equivalent throughout the simulation (Figure 5D). Between 80 and 100 ns, the mean hydrogen bond counts were 263.99 ± 7.39 for the thymol complex and 263.89 ± 8.79 for the p-cymene complex, indicating that both systems maintained a highly similar polar interaction profile. Since the difference between the two complexes was negligible, the divergence observed in radius of gyration is more plausibly related to differences in hydrophobic packing than to changes in hydrogen bond stabilization. In the initial 0 to 20 ns interval, the thymol complex exhibited slightly lower hydrogen bond counts than the p-cymene complex before both systems converged. This pattern accompanied the early variation in radius of gyration and suggests a transient solvent-mediated reorganization during the initial compaction phase. These MD results are mechanistically consistent with the preceding docking analysis. In docking, thymol and p-cymene achieved closely comparable ChemPLP scores and interacted with cathepsin L active-site residues exclusively through hydrophobic contacts, without forming conventional hydrogen bonds. Molecular dynamics simulations reinforced this interpretation by showing that both compounds remained associated with the protein over the nanosecond timescale, but neither produced a dynamic pattern consistent with strong and specific occupation of the catalytic site. In particular, no persistent polar interactions were established with the catalytic dyad Cys25 and His163 [53,54], and no evident restriction of active site loop mobility was observed during the simulations. The lack of sustained anchoring to catalytic residues, together with the preservation of loop flexibility, does not support a selective inhibitory mechanism. Instead, the interaction profile is more consistent with predominantly hydrophobic and nonspecific binding, in agreement with the broad spectrum rather than target-selective toxicity commonly reported for plant monoterpenes in arthropods [55,56]. In this context, the acaricidal activity of TVEO nanoemulsion against *T. mexicanus* is more plausibly related to these general lipophilic effects than to direct inhibition of cathepsin L. Accordingly, the docking results obtained for cathepsin L should be interpreted as part of a broader target screening strategy, rather than as evidence of a well-defined single target mode of action.

## 3. Materials and Methods

### 3.1. Plant Material and Essential Oil Extraction

Fresh *Thymus vulgaris* plants were purchased at the Municipal Market Adolpho Lisboa (Manaus, AM, Brazil) in 2024. Botanical identity was confirmed by comparison with voucher specimen No. RFA2168, deposited at the Herbarium of the Department of Botany, Institute of Biology-UFAM.

Approximately 200 g of fresh, fragmented leaves underwent hydrodistillation with 3 L of water for 2 h using a modified Clevenger-type apparatus [57]. Yield was calculated as percentage weight of essential oil relative to fresh leaf mass (% *w*/*w*). Until use, the essential oil was stored in amber glass bottles under refrigeration to minimize oxidation and volatilization losses.

### 3.2. Preparation of Nanoemulsion and Particle Characterization

TVEO-NE was prepared via the low-energy phase-inversion method described by Ostertag et al. [58]. Briefly, ultrapure water was weighed into a glass vessel, and Tween 20 (Sigma) was combined with *T. vulgaris* essential oil (TVEO) on an analytical balance to achieve a final mass of 2 g (10% surfactant + EO: 90% ultrapure water, *w*/*w*). The formulation presented an HLB of 16.7, corresponding to the intrinsic value of Tween 20, a condition consistent with the production of physically stable oil-in-water dispersions. A corresponding blank nanoemulsion (Blank NE) was prepared under the same conditions, but without TVEO, and used as a vehicle-only control to account for potential effects of the excipients.Particle size and polydispersity index (PDI) were determined in triplicate by photon correlation spectrometry (Litesizer, Anton Paar, Austria). Zeta potential was measured via electrophoretic mobility on the same instrument.

### 3.3. Analytical Methods

Chemical profiling of TVEO employed gas chromatography coupled to mass spectrometry (GC–MS). Analyses were performed on an Agilent 6890N GC interfaced with an Agilent 5973N quadrupole mass spectrometer (Agilent Technologies, Santa Clara, CA, USA) operating in electron-impact mode (70 eV). Chromatographic separation was performed using a DB-5MS capillary column (30 m × 0.25 mm i.d.; 0.25 μm film thickness). Samples (1 μL) were injected in splitless mode; the oven temperature ramped from 40 °C to 300 °C at 4 °C/min. Helium served as carrier gas (0.5 mL/min); injector and ion-source temperatures were maintained at 250 °C and 230 °C, respectively. Mass spectra were acquired over 40–700 Da.

Compound identification combined three approaches: matching of mass-spectral fragmentation patterns with the Wiley NBS database, comparison of calculated retention indices with published values [59], and, where available, co-injection of authentic standards.

### 3.4. Mite Colony

Adult *T. mexicanus* served as the target organism. Stock colonies were maintained at the Laboratory of Acarology and Agricultural Entomology, Federal University of Amazonas (UFAM), Itacoatiara-AM, Brazil, on detached papaya leaves (*Carica papaya* L.), following the rearing protocol of Barroncas et al. [5] and Amaral et al. [60]. Environmental conditions in the climate-controlled chamber were held at 25 °C, 55% relative humidity, and a 12:12 h (L:D) photoperiod.

### 3.5. Bioassay with Mites

Nanoformulations were applied by microspraying, adapting the protocols of Mascarin et al. [61], Spence et al. [62], and Apolinario et al. 2025 [63]. A precision airbrush (Vonder, model 6220016000; 0.3 mm nozzle) connected to a compressor (SOLAB, model SL 61) delivered treatments at 15 psi, with the nozzle fixed 30 cm from the target surface and an application time of 4 s per disc.

The deposited volume per unit area was determined by gravimetric calibration under the exact operational conditions described above. Calibration surfaces (18 × 18 mm; area: 3.24 cm^2^) were weighed individually on an analytical balance (resolution: 0.1 mg) before and immediately after spraying (n = 11); deposited mass was converted to volume assuming a density of 1.00 g/mL for the aqueous nanoemulsion. The mean deposited volume of the spray mixture was 2.91 ± 0.15 µL per 3.24 cm^2^ (CV = 5.2%). Scaled proportionally to the papaya leaf disc area (32.5 mm diameter; 8.30 cm^2^), the estimated deposited volume of nanoemulsion per disc was 7.45 µL (95% CI: 7.19–7.71 µL), containing 223.6 µg of active ingredient (26.9 µg a.i./cm^2^) at the spray concentration of 30.0 mg a.i./mL.

Spray mixtures were prepared at two concentrations: 0.0 (untreated control) and 30.0 mg a.i./mL. Each dilution was vortexed (2800 rpm, 30 s) in a Falcon tube immediately before application to ensure homogeneity.

Contact toxicity was evaluated on papaya leaf discs (32.5 mm diameter), an exposure system that likely combined cuticular and oral routes, since mites feeding on treated discs may co-ingest nanoemulsion droplets deposited on the adaxial leaf surface [50]. Ten adult *T. mexicanus* females, transferred under a stereomicroscope using a fine-bristle brush, were confined on each disc. Discs rested on moistened filter paper (4 cm diameter) atop a polyethylene sponge (5 cm diameter × 1 cm thick) inside a Petri dish (6 cm diameter × 2 cm height). Sponges remained saturated with distilled water throughout the assay; hydrophilic cotton, also moistened, bordered each disc to maintain leaf turgidity and prevent mite escape [5].

Experimental units were held on a laboratory bench under ambient conditions: mean temperature 26.0 ± 2.54 °C, mean relative humidity 55.0 ± 10.83%, and an approximate 12 h photophase provided by natural daylight supplemented with artificial illumination. At 24 h intervals over two consecutive days, units were examined stereoscopically, recording the numbers of live females, dead females, and eggs laid.

### 3.6. In Silico Analysis

#### 3.6.1. Sequence Retrieval and Homology Modeling

The amino acid sequence of cathepsin L from *T. mexicanus* (UniProt accession T1KVN5; 351 residues) was retrieved from the UniProtKB database. Three comparative homology modeling approaches were employed: SWISS-MODEL (https://swissmodel.expasy.org/, accessed on 14 February 2026) [64], I-TASSER (https://zhanggroup.org/I-TASSER/, accessed on 14 February 2026) [65], and Phyre2 (http://www.sbg.bio.ic.ac.uk/phyre2/, accessed on 15 February 2026) [66].

#### 3.6.2. Model Quality Assessment

Structural validation and model quality assessment were performed using complementary tools. Stereochemical quality was evaluated with MolProbity (via the SWISS-MODEL Structure Assessment), reporting MolProbity score, clashscore, Ramachandran favored/outliers, rotamer outliers, and Cβ deviations [45]. Global and local model quality were assessed using QMEAN/QMEANDisCo (QMEAN6, corresponding Z-scores, and residue-level local quality estimates) [47,48]. Independent global validation was performed with ProSA-web, with Z-scores interpreted in the context of experimentally determined structures of similar size rather than as an absolute cutoff [46].

#### 3.6.3. Selection and Construction of 3D Ligand Structures

Three-dimensional (3D) structure files for thymol (PubChem CID: 6989), p-cymene (PubChem CID: 7463), and E64 (PubChem CID: 5742832) were retrieved from the PubChem Compound database in SDF format, selecting the pre-computed 3D conformer. All structures were subjected to energy minimization using the MMFF94 force field as implemented in Avogadro (version 1.2.0), employing the steepest descent algorithm. The optimized 3D structures were exported in mol2 format for subsequent docking analyses.

#### 3.6.4. Binding Site Identification and Molecular Docking

Binding site identification was performed using the CB-Dock2 server (http://cadd.labshare.cn/cb-dock2/, accessed on 3 April 2026) in Auto Blind Docking mode. CB-Dock2 identified five major cavities (C1–C5). Cavity C4 (volume 135 Å^3^, center coordinates x = −13, y = 7, z = 12) was selected based on Vina scoring and structural criteria.

Focused molecular docking was then performed using GOLD v2024.3.0 (Cambridge Crystallographic Data Centre) with the ChemPLP (Piecewise Linear Potential) scoring function. The binding site was defined as a 10.0 Å radius sphere centered on the C4 cavity coordinates (x = −13, y = 7, z = 12). Docking calculations were carried out with default genetic algorithm parameters, automatic early termination criteria, and cavity detection enabled. The top 10 binding poses were generated for each ligand and ranked based on ChemPLP fitness scores. Binding mode convergence was assessed by calculating the amplitude (ΔFitness) across all generated poses for each ligand, defined as ΔFitness = max (PLP.Fitness) − min (PLP.Fitness). Convergence threshold was set at ΔFitness < 2.0.

#### 3.6.5. Analysis of Intermolecular Interactions and Figure Construction

Molecular docking results and receptor–ligand interaction poses were analyzed and visualized using Discovery Studio Visualizer v21.1.0.20298 (BIOVIA). The best binding poses, selected based on the highest ChemPLP fitness scores, were examined for key intermolecular interactions including hydrogen bonds (distance < 3.5 Å, angle > 120°), hydrophobic contacts (alkyl and π-alkyl interactions), and electrostatic interactions. Two-dimensional interaction diagrams were generated for each ligand–receptor complex.

### 3.7. Data Analysis

Laboratory efficacy trials followed a completely randomized design arranged as a 3 × 3 factorial: three exposure times (24, 48, and 72 h) crossed with three treatments (TVEO-NE, Blank-NE, and untreated control), each replicated ten times.

Cumulative female mortality at each evaluation was converted to corrected mortality using the Schneider–Orelli [67] formula: Corrected Mortality (%) = 100 × [(Mortality_(treatment) − Mortality_(control))/(100 − Mortality_(control))].

Daily fecundity was calculated by dividing the number of eggs laid in each 24 h interval by the number of live females at that evaluation. Fertility inhibition (sublethal effect) was derived as: Fertility Inhibition (%) = 100 × [(Fecundity_(control) − Fecundity_(treatment))/Fecundity_(control)].

Prior to parametric analysis, proportional data were transformed to satisfy normality and homoscedasticity assumptions. The Box–Cox procedure [68] indicated λ = 1/2 for both corrected mortality and fecundity inhibition; therefore, both variables were square-root transformed as [(x/100) + 0.5]^(1/2) before ANOVA. Transformed data were subjected to factorial ANOVA, and treatment means were separated by the Scott-Knott test [69] at α = 0.05. All analyses were performed in R 4.0.0 (R Core Team, 2020; R Foundation for Statistical Computing, Vienna, Austria) using the packages asbio, BioStatR, comperes, easy Anova, and MASS.

## 4. Conclusions

A monoterpene-rich nanoemulsion containing *Thymus vulgaris* essential oil was produced by low-energy emulsification and displayed physicochemical properties suitable for contact bioassays. The formulation produced clear lethal and sublethal effects against adult *T. mexicanus*, whereas the blank nanoemulsion induced only limited responses, indicating that the acaricidal activity was mainly driven by the essential oil. Morphological examination of treated colonies revealed structural disruption consistent with membrane-partitioning mode of action, though direct experimental confirmation remains to be established. Molecular docking and molecular dynamics simulations against a cathepsin L homology model of *T. mexicanus* did not support specific cysteine protease inhibition as the primary mode of action, as the principal oil constituents thymol and p-cymene showed lower predicted binding than E64 and lacked interactions compatible with catalytic-site inhibition. Overall, these findings position the developed monoterpene-based nanoemulsion as a promising botanical tool for the management of *T. mexicanus* and highlight membrane-related disruption as a plausible mechanistic pathway for future studies. 

## Figures and Tables

**Table 1:** Chemical composition of the essential oil of *Thymus vulgaris* analyzed by gas chromatography–mass spectrometry.

Compounds_(CAS)_	RI Lit. ^a^	RI Cal. ^b^	Area (%)
α-pinene_80-56-8_	932	933	0.34
camphene_79-92-5_	946	942	0.21
1-octen-3-ol_3391-86-4_	972	973	0.66
eucalyptol_470-82-6_	1026	1025	1.06
γ-terpinene_99-85-4_	1054	1052	1.0
p-cymene_99-87-6_	1089	1089	37.0
linalool_78-70-6_	1095	1094	1.69
endo-borneol_507-70-0_	1155	1153	1.18
terpinen-4-ol_562-74-3_	1174	1174	1.0
α-terpineol_98-55-5_	1186	1185	0.45
thymyl methyl ether_1076-56-8_	1232	1233	1.23
D-carvone_2244-16-8_	1239	1238	0.59
carvacrol methyl ether_6379-73-3_	1241	1241	0.82
thymol_89-83-8_	1289	1287	45.86
4-isopropyl-3-methylphenol_3228-02-2_	1332	1330	4.30
caryophyllene_87-44-5_	1417	1420	0.48
caryophyllene oxide_1139-30-6_	1582	1580	0.69
Total			98.56

^a^ Retention index from literature. ^b^ Retention index based on a homologous series of normal alkanes.

**Table 2:** Composition and physicochemical characterization of *Thymus vulgaris* essential oil nanoemulsion (TVEO-NE), and corresponding blank nanoemulsion (Blank-NE).

Parameter	TVEO-NE	Blank-NE
Tween 20 (g)	0.0512	0.0512
Essential oil, TVEO (g)	0.0509	0.0000
Ultrapure water (g)	0.9047	0.9556 *
HLB	16.7	16.7
Particle size (nm)	200.70 ± 8.9	209.4 ± 2.7
Polydispersity index (PDI)	0.237	0.245
Zeta potential (mV)	−22.2 ± 0.9	−20.1 ± 0.6

* Blank-NE water calculated by replacing the essential-oil mass with water (constant total mass). Abbreviations: EO, essential oil; HLB, hydrophilic–lipophilic balance.

**Table 3:** Comparative evaluation of 3D structural models obtained using three different approaches.

Model	MolProbity Score	Clash Score	Ramachan-Dran Favored (%)	Ramachan-Dran Outliers (%)	Rotamer Outliers (%)	Cβ Deviations (n)	QMEAN6	Z-QMEAN	ProSA Z-Score
SWISS-MODEL	1.49	0.37	92.84	2.29	3.33	4	0.734	−1.06	−7.57
Phyre2.2	2.58	50.08	93.97	1.27	0.00	0	0.734	−1.06	−7.79
I-TASSER	5.11	361.45	76.22	9.17	54.33	313	0.439	−8.32	−8.09

Notes: Ramachandran statistics were obtained from MolProbity (Structure Assessment). QMEAN6 corresponds to the normalized QMEAN6 global score and Z-QMEAN to its z-score. ProSA Z-scores are maintained as previously reported.

**Table 4:** Molecular docking results for ligand–cathepsin L interactions (GOLD Suite 2024.3.0, ChemPLP scoring function). Best PLP.Fitness score, key binding site residues, and hydrogen bond contribution (PLP.Chemscore.Hbond) are reported for the highest-ranked pose of each ligand–receptor complex. E64 was included as a positive control inhibitor.

Ligand	Best PLP.Fitness	Key Interacting Residues	H-Bond Score (Best Pose)
E64 (control)	63.84	GLU A:276, GLU A:288, GLU A:290, SER A:277, PRO A:289, HIS A:89	5.38
Tween 20	69.20	ARG A:79, ARG A:83, ILE A:82, LYS A:76, GLU A:276, SER A:277, HIS A:103, SER A:274, GLU A:290	2.25
Thymol	36.13	HIS A:80, HIS A:89, LYS A:76, LEU A:93	0.00
p-Cymene	35.46	ARG A:79, HIS A:80, LEU A:93	0.00

## Data Availability

The data that support the findings of this study are available from the corresponding author upon reasonable request.

## Supplementary Materials

The following supporting information can be downloaded at: https://www.mdpi.com/article/10.3390/molecules31122167/s1, Table S1. Two-way ANOVA (3 × 3) for corrected mortality of adult females of Tetranychus mexicanus (TVEO-NE). Table S2. Two-way ANOVA (3 × 3) for fecundity inhibition of Tetranychus mexicanus (TVEO-NE). Figure S1. Intermolecular interactions of control ligands with cathepsin L from Tetranychus mexicanus. Left panel: three-dimensional structure of cathepsin L with the binding site region highlighted (space-filling representation). (A,B) E64 (positive control): (A) three-dimensional view of the ligand–cathepsin L complex; (B) two-dimensional diagram of intermolecular interactions. (C,D) Tween 20 structural surrogate (PubChem CID 443314): (C) three-dimensional binding pose; (D) two-dimensional interaction diagram. Interaction legend: conventional hydrogen bonds (green), carbon-hydrogen bonds (light green), alkyl interactions (light pink), and π-alkyl interactions (pink). Visualizations generated with BIOVIA Discovery Studio 2021.

## References

[1] Mirza J.H., Kamran M., Alatawi F.J. (2018). Webbing life type and behavioral response of the date palm mite, *Oligonychus afrasiaticus*, to webbing residues on leaves and fruits of date palm. Exp. Appl. Acarol..

[2] Hong P., Dash C.K., Ghafar M.A., Haq I.U., Lu L., Zhou C., Wu Q., Wang L. (2024). Demography and population projection of *Tetranychus urticae* (Tetranychidae) on *Phaseolus vulgaris* (Fabaceae) colonized by entomopathogenic fungal endophytes. Insects.

[3] Saito Y., Sato Y. (2024). Diversity in life types of spider mites. Front. Arachn. Sci..

[4] Chiaradia L.A., Milanez J.M., Nesi C.N. (2009). Influência de fatores climáticos e de ácaros predadores na população de ácaros tetraniquídeos em citros. Agropec. Catarin..

[5] Barroncas J.F., Silva N.M., Vasconcelos G.J.N. (2022). Biologia de *Tetranychus mexicanus* (McGregor) (Acari: Tetranychidae) em mamoeiro e maracujazeiro. Pesq. Agropec. Trop..

[6] Santos R.S., Ferla N.J., Ferla J.J., Silva W. (2018). Record of *Tetranychus mexicanus* (McGregor) (Acari: Tetranychidae) in papaya plant (*Carica papaya* L.) in the Acre State, Brazil. EntomoBrasilis.

[7] El-Sayed M.A., El-Gohary L.R., Saleh A., Said A. (2023). Effectiveness of plant extracts and traditional compounds against *Tetranychus urticae* Koch, under field conditions in squash. J. Plant Prot. Pathol..

[8] Susurluk H. (2023). Potential use of essential oils from *Origanum vulgare* and *Syzygium aromaticum* to control *Tetranychus urticae* Koch (Acari: Tetranychidae) on two host plant species. PeerJ.

[9] Van Leeuwen T., Vontas J., Tsagkarakou A., Dermauw W., Tirry L. (2010). Acaricide resistance mechanisms in the two-spotted spider mite *Tetranychus urticae* and other important Acari: A review. Insect Biochem. Mol. Biol..

[10] Jakubowska M., Dobosz R., Zawada D., Kowalska J. (2022). A review of crop protection methods against the twospotted spider mite—*Tetranychus urticae* Koch (Acari: Tetranychidae)—With special reference to alternative methods. Agriculture.

[11] De Rouck S., İnak E., Dermauw W., Van Leeuwen T. (2023). A review of the molecular mechanisms of acaricide resistance in mites and ticks. Insect Biochem. Mol. Biol..

[12] Attia S., Grissa K.L., Lognay G., Bitume E., Hance T., Mailleux A.C. (2013). A review of the major biological approaches to control the worldwide pest *Tetranychus urticae* (Acari: Tetranychidae) with special reference to natural pesticides. J. Pest Sci..

[13] Alfaro-Valle E., Martínez-Hernández A., Otero-Colina G., Lara-Reyna J. (2022). High susceptibility of *Tetranychus merganser* (Acari: Tetranychidae), an emergent pest of the tropical crop *Carica papaya*, towards *Metarhizium anisopliae* s.l. and *Beauveria bassiana* strains. PeerJ.

[14] Fidelis de Santana M., Ferla N.J., Dionisio L.F.S., de Lima Santos N., da Silva Pedreira L.H., Poltronieri A.S. (2021). Bioactivity of essential oils for the management of *Tetranychus urticae* Koch and selectivity on its natural enemy *Neoseiulus californicus* (McGregor). Acarologia.

[15] Bakkali F., Averbeck S., Averbeck D., Idaomar M. (2008). Biological effects of essential oils—A review. Food Chem. Toxicol..

[16] de Sousa D.P., Damasceno R.O.S., Amorati R., Elshabrawy H.A., de Castro R.D., Bezerra D.P., Nunes V.R.V., Gomes R.C., Lima T.C. (2023). Essential oils: Chemistry and pharmacological activities. Biomolecules.

[17] Pavela R., Benelli G. (2016). Essential oils as ecofriendly biopesticides? Challenges and constraints. Trends Plant Sci..

[18] Isman M.B. (2020). Botanical insecticides in the twenty-first century—Fulfilling their promise?. Annu. Rev. Entomol..

[19] Musa A., Međo I., Marić I., Marčić D. (2017). Acaricidal and sublethal effects of a *Chenopodium*-based biopesticide on the two-spotted spider mite (Acari: Tetranychidae). Exp. Appl. Acarol..

[20] Badawy M.E.I., Abdelgaleil S.A.M., Mahmoud N.F., Marei A.E.-S.M. (2018). Preparation and characterizations of essential oil and monoterpene nanoemulsions and acaricidal activity against two-spotted spider mite (*Tetranychus urticae* Koch). Int. J. Acarol..

[21] Mossa A.H., Afia S.I., Mohafrash S.M.M., Abou-Awad B.A. (2019). Rosemary essential oil nanoemulsion, formulation, characterization and acaricidal activity against the two-spotted spider mite *Tetranychus urticae* Koch (Acari: Tetranychidae). J. Plant Prot. Res..

[22] Ribeiro N.C., Camara C.A.G., Melo J.P.R., Moraes M.M. (2019). Effect of the essential oil from the latex of the fruit *Mangifera indica* L. on *Tetranychus urticae* Koch (Acari, Tetranychidae). Acarologia.

[23] An H., Tak J.-H. (2022). Miticidal and repellent activity of thirty essential oils and their synergistic interaction with vanillin against *Tetranychus urticae* Koch (Acari: Tetranychidae). Ind. Crops Prod..

[24] Ebadollahi A., Ziaee M., Palla F. (2020). Essential oils extracted from different species of the Lamiaceae plant family as prospective bioagents against several detrimental pests. Molecules.

[25] Modarres-Najafabadi S.S., Taji M., Hajihassani A. (2012). Study on *Thymus vulgaris*, *Lavandula officinalis* and *Eucalyptus camaldulensis* extracts on the two-spotted spider mite. Int. J. AgriScience.

[26] Wu L., Huo X., Zhou X., Zhao D., He W., Liu S., Liu H., Feng T., Wang C. (2017). Acaricidal activity and synergistic effect of thyme oil constituents against carmine spider mite (*Tetranychus cinnabarinus* (Boisduval)). Molecules.

[27] Hammoudi Halat D., Krayem M., Khaled S., Younes S. (2022). A focused insight into thyme: Biological, chemical, and therapeutic properties of an indigenous Mediterranean herb. Nutrients.

[28] Gupta P., Preet S., Ananya, Singh N. (2022). Preparation of *Thymus vulgaris* (L.) essential oil nanoemulsion and its chitosan encapsulation for controlling mosquito vectors. Sci. Rep..

[29] Turek C., Stintzing F.C. (2013). Stability of essential oils: A review. Compr. Rev. Food Sci. Food Saf..

[30] Alibeigi Z., Rakhshandehroo E., Saharkhiz M.J., Alavi A.M. (2025). The acaricidal and repellent activity of the essential and nano essential oil of *Thymus vulgaris* against the larval and engorged adult stages of the brown dog tick, *Rhipicephalus sanguineus* (Acari: Ixodidae). BMC Vet. Res..

[31] Satyal P., Murray B.L., McFeeters R.L., Setzer W.N. (2016). Essential oil characterization of *Thymus vulgaris* from various geographical locations. Foods.

[32] Patil S.M., Ramu R., Shirahatti P.S., Shivamallu C., Amachawadi R.G. (2021). A systematic review on ethnopharmacology, phytochemistry and pharmacological aspects of *Thymus vulgaris* Linn. Heliyon.

[33] Németh-Zámboriné É., Preedy V.R. (2016). Natural variability of essential oil components. Essential Oils in Food Preservation, Flavor and Safety.

[34] Gholamzadeh-Chitgar M., Khosravi R., Jalali-Sendi J., Ghadamyari M. (2013). Sublethal effects of *Thymus vulgaris* essential oil on life-table parameters of two-spotted spider mite, *Tetranychus urticae* Koch (Acari: Tetranychidae). Arch. Phytopathol. Plant Prot..

[35] Pavoni L., Perinelli D.R., Bonacucina G., Cespi M., Palmieri G.F. (2020). An overview of micro- and nanoemulsions as vehicles for essential oils: Formulation, preparation and stability. Nanomaterials.

[36] Al-Asmary F., Koirala P., Rathod N.B., Alnemr T.M., Asiri S.A., Babeker M.Y., Li L., Nirmal N.P. (2024). Antimicrobial activity of formulated *Origanum* and thyme essential oil nanoemulsions—A comparative study. Curr. Nutr. Food Sci..

[37] Zhao S., Wang Z., Wang X., Kong B., Liu Q., Xia X., Liu H. (2023). Characterization of nanoemulsions stabilized with different emulsifiers and their encapsulation efficiency for oregano essential oil: Tween 80, soybean protein isolate, tea saponin, and soy lecithin. Foods.

[38] Pavoni L., Pavela R., Cespi M., Bonacucina G., Maggi F., Zeni V., Canale A., Lucchi A., Bruschi F., Benelli G. (2019). Green Micro- and Nanoemulsions for Managing Parasites, Vectors and Pests. Nanomaterials.

[39] Stepanycheva E.A., Petrova M.O., Chermenskaya T.D. (2023). Biological activity of *Litsea cubeba* essential oil and citral against the two-spotted spider mite *Tetranychus urticae* Koch (Acarina, Tetranychidae). Entomol. Rev..

[40] Abouelmaaty H.G., Fukushi M., Abouelmaaty A.G., Ghazy N.A., Suzuki T. (2019). Leaf disc-mediated oral delivery of small molecules in the absence of surfactant to the two-spotted spider mite, *Tetranychus urticae*. Exp. Appl. Acarol..

[41] Desneux N., Decourtye A., Delpuech J.-M. (2007). The sublethal effects of pesticides on beneficial arthropods. Annu. Rev. Entomol..

[42] McEnroe W.D. (1961). Guanine excretion by the two-spotted spider mite (*Tetranychus telarius* (L.)). Ann. Entomol. Soc. Am..

[43] Wiesmann R. (1968). Untersuchungen über die Verdauungsvorgänge bei der gemeinen Spinnmilbe, *Tetranychus urticae* Koch. Z. Angew. Entomol..

[44] Mothes-Wagner U. (1985). Fine structure of the ‘hindgut’ of the two-spotted spider mite, *Tetranychus urticae*, with special reference to origin and function. Exp. Appl. Acarol..

[45] Chen V.B., Arendall W.B., Headd J.J., Keedy D.A., Immormino R.M., Kapral G.J., Murray L.W., Richardson J.S., Richardson D.C. (2010). MolProbity: All-atom structure validation for macromolecular crystallography. Acta Crystallogr. D. Biol. Crystallogr..

[46] Wiederstein M., Sippl M.J. (2007). ProSA-web: Interactive web service for the recognition of errors in three-dimensional structures of proteins. Nucleic Acids Res..

[47] Benkert P., Biasini M., Schwede T. (2011). Toward the estimation of the absolute quality of individual protein structure models. Bioinformatics.

[48] Studer G., Rempfer C., Waterhouse A.M., Gumienny R., Haas J., Schwede T. (2020). QMEANDisCo—Distance constraints applied on model quality estimation. Bioinformatics.

[49] Waterhouse A., Bertoni M., Bienert S., Studer G., Tauriello G., Gumienny R., Heer F.T., de Beer T.A.P., Rempfer C., Bordoli L. (2018). SWISS-MODEL: Homology modelling of protein structures and complexes. Nucleic Acids Res..

[50] Bensoussan N., Zhurov V., Yamakawa S., O’Neil C.H., Suzuki T., Grbić M., Grbić V. (2018). The digestive system of the two-spotted spider mite, *Tetranychus urticae* Koch, in the context of the mite–plant interaction. Front. Plant Sci..

[51] Santamaría M.E., González-Cabrera J., Martínez M., Grbic V., Castañera P., Díaz L., Ortego F. (2015). Digestive proteases in bodies and faeces of the two-spotted spider mite, *Tetranychus urticae*. J. Insect Physiol..

[52] El-Sayed S.M., Ahmed N., Selim S., Al-Khalaf A.A., El Nahhas N., Abdel-Hafez S.H., Sayed S., Emam H.M., Ibrahim M.A.R. (2022). Acaricidal and antioxidant activities of anise oil (*Pimpinella anisum*) and the oil’s effect on protease and acetylcholinesterase in the two-spotted spider mite (*Tetranychus urticae* Koch). Agriculture.

[53] Turk V., Stoka V., Vasiljeva O., Renko M., Sun T., Turk B., Turk D. (2012). Cysteine cathepsins: From structure, function and regulation to new frontiers. Biochim. Biophys. Acta Proteins Proteom..

[54] Mondragón-Suárez A.K., Martí S., Moliner V. (2023). Impact of the warhead of dipeptidyl keto Michael acceptors on the inhibition mechanism of cysteine protease cathepsin L. ACS Catal..

[55] Isman M.B. (2006). Botanical insecticides, deterrents, and repellents in modern agriculture and an increasingly regulated world. Annu. Rev. Entomol..

[56] Regnault-Roger C., Vincent C., Arnason J.T. (2012). Essential oils in insect control: Low-risk products in a high-stakes world. Annu. Rev. Entomol..

[57] Brazil (2019). Brazilian Pharmacopoeia.

[58] Ostertag F., Weiss J., McClements D.J. (2012). Low-energy formation of edible nanoemulsions: Factors influencing droplet size produced by emulsion phase inversion. J. Colloid Interface Sci..

[59] Adams R. (2009). Identification of Essential Oil Components by Gas Chromatography/Mass Spectrometry.

[60] Amaral A.C.F., Ramos A.S., Pena M.R., Ferreira J.L.P., Menezes J.M.S., Vasconcelos G.J.N., Silva N.M., Silva J.R.A. (2017). Acaricidal activity of *Derris floribunda* essential oil and its main constituent. Asian Pac. J. Trop. Biomed..

[61] Mascarin G.M., Quintela E.D., Silva E.G., Arthurs S.P. (2013). Precision micro-spray tower for application of entomopathogens. BioAssay.

[62] Spence E.L., Chandler D., Edgington S., Berry S.D., Martin G., O’Sullivan C., Svendsen C., Hesketh H. (2020). A standardised bioassay method using a bench-top spray tower to evaluate entomopathogenic fungi for control of the greenhouse whitefly, Trialeurodes vaporariorum. Pest Manag. Sci..

[63] Apolinário R.V.C., Cruz J.D., Neto W.S.M.F., Soares J.M.C., Mpalantinos M.A., Gomes S.A.O., Feder D., Ferreira J.L.P., Vasconcelos G.J.N., Silva J.R.A. (2025). Insecticidal and Ovicidal Activity of Cymbopogon citratus Essential Oil and Its Nanoemulsion Against Hemipteran Crop Pests with Mortality, Antennal Malformations, and Volatile Alterations. Insects.

[64] Biasini M., Bienert S., Waterhouse A., Bertoni M., Bordoli L., Studer G., Tiwari S., Schwede T. (2014). SWISS-MODEL: Modelling protein tertiary and quaternary structure using evolutionary information. Nucleic Acids Res..

[65] Zhang Y. (2008). I-TASSER server for protein 3D structure prediction. BMC Bioinform..

[66] Powell H.R., Islam S.A., David A., Sternberg M.J.E. (2025). Phyre2.2: A community resource for template-based protein structure prediction. J. Mol. Biol..

[67] Schneider-Orelli O. (1947). Entomologisches Praktikum — Einführung in die land- und forstwirtschaftliche Insektenkunde.

[68] Box G.E.P., Cox D.R. (1964). An analysis of transformations. J. R. Stat. Soc. Ser. B.

[69] Scott A.J., Knott M. (1974). A cluster analysis method for grouping means in the analysis of variance. Biometrics.

