# Supplementary material for: Monoterpene-Rich Nanoemulsion from Thymus vulgaris as a Promising Acaricidal Strategy Against Tetranychus mexicanus: Effects on Survival and Fecundity
Source: Molecules. 2026 Jun 20;31(12):2167. doi: 10.3390/molecules31122167 (PMC13306013; doi:10.3390/molecules31122167)
Supplement: Supplementary file 1 [file molecules-31-02167-s001.zip › molecules-4359323-supplementary.pdf]

Monoterpene-Rich Nanoemulsion from *Thymus vulgaris* as a Promising Acaricidal strategy against *Tetranychus mexicanus*: Effects on Survival and Fecundity

Geraldo J. N. Vasconcelos[1], Raul V. C. Apolinário[2], Tatiane M. S. Cardoso[1], Jefferson D. Cruz[2], Walter S. M. F. Neto[3], Maria A. Mpalantinos[2], Jefferson R. A. Silva[3*], Ana Claudia F. Amaral[2*]

[1] Universidade Federal do Amazonas, ICET, Itacoatiara 69103-128, AM, Brazil; gjnvasconcelos@ufam.edu.br

[2] Laboratório de Plantas Medicinais e Derivados, Farmanguinhos, Fundação Oswaldo Cruz, Rio de Janeiro 21041-250, RJ, Brazil; apolinario_raul@yahoo.com (R.V.C.A.); jefferson_dacruz@hotmail.com (J.D.C.); maria.mpalantinos@fiocruz.br (M.A.M.)

[3] Laboratório de Cromatografia, Departamento de Química, Instituto de Ciências Exatas, Universidade Federal do Amazonas, Manaus 69077-000, AM, Brazil; wssottoo@gmail.com (W.S.M.F.N.)

* Correspondence: jrocha_01@yahoo.com.br (J.R.A.S.); aamaral_99@yahoo.com.br (A.C.F.A.)

Table S1. Two-way ANOVA (3 × 3) for corrected mortality of adult females of *Tetranychus mexicanus* (TVEO-NE).

**Source of variation**	**TVEO-NE**				
	**df**	**Type III SS**	**Mean square**	**F**	***p*-value**
**Exposure factor (FE)**	2	0.2435	0.1218	8.4706	< 0.001
**Formulation factor (FF)**	2	0.7668	0.3834	26.6713	< 0.001
**FE × FF**	4	0.2861	0.0715	4.9758	0.0012
**Residual**	81	1.1644	0.0144	--	--
**Shapiro–Wilk p-value**					0.0002
**Bartlett's test p-value (FE)**					0.0034
**Bartlett's test p-value (FF)**					0.0025
**Coefficient of variation (%)**					14.89

Note: Data were transformed using [(x/100) + 0.5]^(1/2). 'ns' = not significant. TVEO-NE = *Thymus vulgaris* essential oil nanoemulsion.

Table S2. Two-way ANOVA (3 × 3) for fecundity inhibition of *Tetranychus mexicanus* (TVEO-NE).

Source of variation	TVEO-NE				
	df	Type III SS	Mean square	F	*p*-value
**Exposure factor (FE)**	2	0.0293	0.0147	0.5687	0.5685 ns
**Formulation factor (FF)**	2	1.2368	0.6184	23.9839	< 0.001
**FE × FF**	4	0.0876	0.0219	0.8492	0.4982 ns
**Residual**	81	2.0884	0.0258	--	--
**Shapiro–Wilk p-value**					0.2858
**Bartlett's test p-value (FE)**					0.8977
**Bartlett's test p-value (FF)**					0.2744
**Coefficient of variation (%)**					19.70

Note: Data were transformed using [(x/100) + 0.5]^(1/2). 'ns' = not significant. TVEO-NE = *Thymus vulgaris* essential oil nanoemulsion.

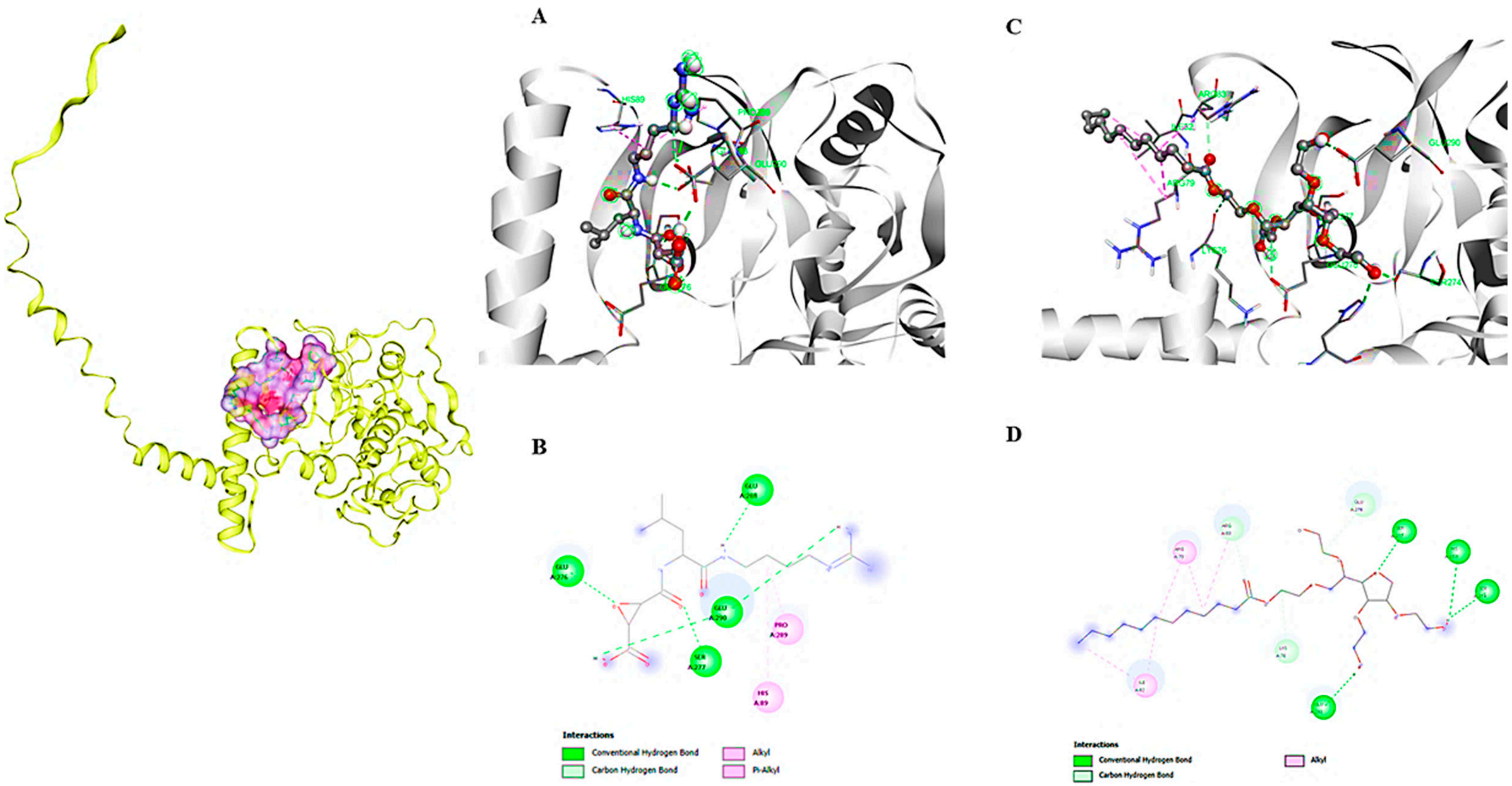

Figure S1. Intermolecular interactions of control ligands with cathepsin L from *Tetranychus mexicanus*. Left panel: three-dimensional structure of cathepsin L with the binding site region highlighted (space-filling representation). **(A, B)** E64 (positive control): **(A)** three-dimensional view of the ligand–cathepsin L complex; **(B)** two-dimensional diagram of intermolecular interactions. **(C, D)** Tween 20 structural surrogate (PubChem CID 443314): **(C)** three-dimensional binding pose; **(D)** two-dimensional interaction diagram. Interaction legend: conventional hydrogen bonds (green), carbon-hydrogen bonds (light green), alkyl interactions (light pink), and π-alkyl interactions (pink). Visualizations generated with BIOVIA Discovery Studio 2021.
